# Delayed accumulation of intestinal coliform bacteria enhances life span and stress resistance in *Caenorhabditis elegans* fed respiratory deficient *E*. *coli*

**DOI:** 10.1186/1471-2180-12-300

**Published:** 2012-12-20

**Authors:** Fernando Gomez, Gabriela C Monsalve, Vincent Tse, Ryoichi Saiki, Emily Weng, Laura Lee, Chandra Srinivasan, Alison R Frand, Catherine F Clarke

**Affiliations:** 1 Molecular Biology Institute, University of California, Los Angeles, CA, 90095, USA; 2 Department of Biological Chemistry, University of California, Los Angeles, CA, 90095, USA; 3 Department of Chemistry and Biochemistry, University of California, Los Angeles, CA, 90095, USA; 4 Department of Chemistry and Biochemistry, California State University, Fullerton, CA, 92834, USA; 5 Present address: C3 Jian, Inc, 4503 Glencoe Ave, Marina del Ray, CA, 90292, USA; 6 Present address: Funakoshi Co., Ltd, 9-7 Hongo 2-Chome, Bunkyo-Ku, Tokyo, 113-0033, Japan; 7 Present address: City Year Los Angeles, 606 South Olive Street, 2nd Floor, Los Angeles, California, 90014, USA

**Keywords:** Aging, Bacterial colonization, Coenzyme Q, Gut microbiome, Intestine, Life span, Pharynx, Probiotic, Respiration

## Abstract

**Background:**

Studies with the nematode model *Caenorhabditis elegans* have identified conserved biochemical pathways that act to modulate life span. Life span can also be influenced by the composition of the intestinal microbiome, and *C*. *elegans* life span can be dramatically influenced by its diet of *Escherichia coli*. Although *C*. *elegans* is typically fed the standard OP50 strain of *E*. *coli*, nematodes fed *E*. *coli* strains rendered respiratory deficient, either due to a lack coenzyme Q or the absence of ATP synthase, show significant life span extension. Here we explore the mechanisms accounting for the enhanced nematode life span in response to these diets.

**Results:**

The intestinal load of *E*. *coli* was monitored by determination of worm-associated colony forming units (cfu/worm or coliform counts) as a function of age. The presence of GFP-expressing *E*. *coli* in the worm intestine was also monitored by fluorescence microscopy. Worms fed the standard OP50 *E*. *coli* strain have high cfu and GFP-labeled bacteria in their guts at the L4 larval stage, and show saturated coliform counts by day five of adulthood. In contrast, nematodes fed diets of respiratory deficient *E*. *coli* lacking coenzyme Q lived significantly longer and failed to accumulate bacteria within the lumen at early ages. Animals fed bacteria deficient in complex V showed intermediate coliform numbers and were not quite as long-lived. The results indicate that respiratory deficient Q-less *E*. *coli* are effectively degraded in the early adult worm, either at the pharynx or within the intestine, and do not accumulate in the intestinal tract until day ten of adulthood.

**Conclusions:**

The findings of this study suggest that the nematodes fed the respiratory deficient *E*. *coli* diet live longer because the delay in bacterial colonization of the gut subjects the worms to less stress compared to worms fed the OP50 *E*. *coli* diet. This work suggests that bacterial respiration can act as a virulence factor, influencing the ability of bacteria to colonize and subsequently harm the animal host. Respiratory deficient bacteria may pose a useful model for probing probiotic relationships within the gut microbiome in higher organisms.

## Background

The digestive tracts of living systems, from nematodes to humans, contain a zoo of microorganisms. Many of these microbiota fill a required role for the host. The microbiota in human gastrointestinal systems produce folate and vitamin K, break down excess sugars and fibers, and help activate certain medications [1,2]. However, digestive tracts also play host to various bacteria associated with pathophysiological states. Ulcerative colitis, diabetes mellitus, and irritable bowel syndrome are just a few of the diseases influenced by intestinal microbiota [1].

Microorganisms of the intestinal tract have been shown to influence the aging process. Metchnikoff suggested that the longevity of Bulgarians was attributed to their consumption of lactic acid generating bacteria in yogurts [3]. Although the composition of the intestinal microbiome seems to be unique to each individual [4], there are common trends when the gut microbiome of babies is compared across diverse cultures [5]. Some studies have shown certain age-related diseases can be prevented or ameliorated with the use of certain microorganisms [6].

Model organisms can be utilized as a first step in assessing the relationship between longevity and the gut microbiome. Altering gut microorganism composition can influence the aging process in model systems in a safe and effective manner [7,8]. Mice fed diets supplemented with *Lactobacillus* as a probiotic not only showed no pathogenic response, but also lived longer than littermates on a standard diet [9].

*C*. *elegans* is routinely maintained on the standard lab *E*. *coli* strain OP50. Wild-type (N2) worms fed this diet live an average of two weeks [10], and recapitulate many of the aging-related changes observed in humans. Old worms show muscular disorganization, diminished movement, and accumulate the aging-related pigment lipofuscin [11,12]. Worms fed OP50 show an accumulation of bacteria in the pharynx and gut as they age [13-15] and old nematodes appear constipated [14]. *C*. *elegans* fed diets of either *Lactobacillus* or *Bifidobacterium* were long-lived and more resistant to the enteropathogen *Salmonella enterica* as compared to worms fed the standard OP50 *E*. *coli* lab diet [16].

Feeding worms a diet of GD1 *E*. *coli* deficient in coenzyme Q (ubiquinone or Q) leads to an increased life span without a cost to fertility [17,18]. Q is an essential lipid component of the electron transport chain and is required for respiration-dependent energy production. The life span increase of nematodes fed a GD1 Q-less *E*. *coli* diet is not due to a lack of Q, because supplying a water-soluble formulation of Q that is effectively assimilated by the worm does not revert the life span of the GD1-fed animals to that observed in OP50-fed animals [18]. In addition, worms fed *E*. *coli* mutant strains with defects in ATP synthase (1100bc or AN120) lived longer than worms fed OP50 [18]. This implied that the respiratory status of the bacteria was a crucial factor in the life span of the worms fed these diets.

The relationship between respiration in the *E*. *coli* diet and the survival of the worms fed these diets identifies Q and ATP synthase as potential virulence factors. A virulence factor is any process, structure or metabolite required by a microorganism to be pathogenic to its host [19]. In this study we show that loss of respiration in *E*. *coli* yields delayed gut colonization and improved worm survival. Indeed, in young animals, few respiratory deficient *E*. *coli* are detected on the posterior side of the pharynx. Worms fed a mixture of Q-replete and Q-deficient *E*. *coli* show intermediate life span extension, indicating that the degree of bacterial colonization of the gut may be dose dependent. We hypothesize that decreased or delayed gut colonization confers a survival advantage to animals fed the Q-deficient *E*. *coli* by diminishing or delaying stress due to high numbers of coliform bacteria. *C*. *elegans* fed respiratory-deficient *E*. *coli* diets serves as a model for characterizing the effects of anti-aging probiotic therapies.

## Results

### The GD1-mediated life span extension is independent of dietary restriction or worm Q content

**Figure 1:**
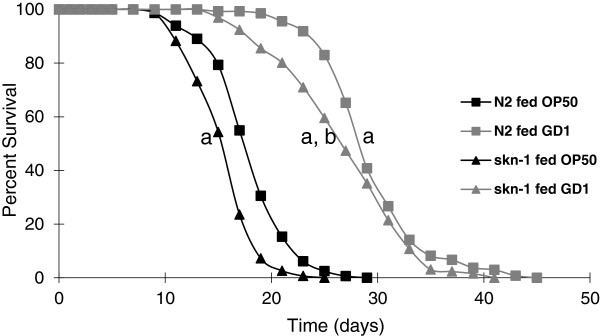
**The oxidative stress sensitive *skn-1(zu169) *mutant, with defects in response to dietary restriction, shows a life span extension in response to the GD1 diet.** Wild-type N2 (squares) and *skn-1(zu169) −/−* mutant worms (triangles) were fed either OP50 (black) (N2, n = 164; *skn-1(zu169) −/−*, n = 153) or GD1 (grey) (N2, n = 135; *skn-1(zu169) −/−*, n = 131) from the L4 stage. N2 worms fed GD1 showed a 67% increase in mean life span as compared to N2 worms fed OP50 (a, p < .0001). *skn-1(zu169) −/−* mutants fed GD1 showed a 50% increase in mean life span compared to N2 worms fed OP50 (a, p < .0001). *skn-1(zu169) −/−* fed OP50 showed an 11% decrease in mean life span as compared to N2 fed OP50 (a, p < .0001). *skn-1(zu169) −/−* fed GD1 showed a 69% increase in mean life span compared to mutants fed OP50 (b, p < .0001). Data were subjected to one-way ANOVA with Fisher’s test at a significance level of p < 0.05.

Findings from previous studies have suggested that the life span increase in *C*. *elegans* fed a Q-less (GD1) *E*. *coli* diet operates independently of dietary restriction [18]. Neither brood size nor worm size, two indicators of dietary restriction, were altered in wild-type animals fed GD1 as compared to the standard OP50 diet [17,18,20]. As a genetic test of the role of dietary restriction, we fed *skn1* mutants the GD1 diet, since these mutants fail to respond to dietary restriction and are sensitive to oxidative stress [21]. SKN-1, a transcription factor homologous to mammalian *Nrf1*, plays a role in metabolic regulation and interacts with signaling systems that respond to changes in nutrition [22]. As shown in Figure 1, *skn1* mutants fed GD1 live longer than hatch-mates fed OP50. These results confirm that the GD1 diet imparts life span extension independently of effects related to dietary restriction.

**Figure 2:**
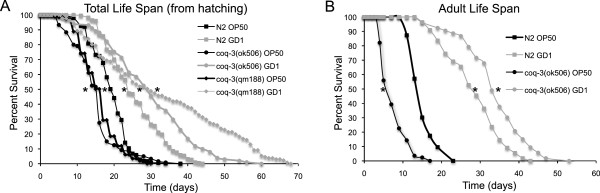
**Q deficient worms respond to GD1 diet.** (**A**) Wild-type (squares), *coq-3(ok506) −/−* (circles) and *coq-3(qm188) −/−* (diamonds) were fed either OP50 (black) (N2, n = 529; *coq-3(ok506) −/−*, n = 119; *coq-3(qm188) −/−*, n = 259) or GD1 (grey) (N2, n = 225; *coq-3(ok506) −/−*, n = 102; *coq-3(qm188) −/−*, n = 141) from the hatchling stage and assessed for survival. *Asterisks* designate: A significant increase in mean life span of N2 fed GD1 compared to OP50: 37% (p < .0001); Increase in mean life span of *coq-3(ok506) −/−* fed GD1 compared to N2 fed OP50: 58% (p < .0001); and Increase in mean life span of *coq-3(qm188) −/−* fed GD1 compared to N2 fed OP50: 74% (p < .0001). (B) Wild-type (squares) and *coq-3(ok506) −/−* (circles) were fed OP50 (black) until the L4 larval stage and then subsequently fed either OP50 (black) (N2, n = 63; *coq-3(ok506) −/−*, n = 84) or GD1 (grey) (N2, n = 55; *coq-3(ok506) −/−*, n = 53) and assessed for survival. Increase in mean life span of N2 worms fed GD1 compared to N2 fed OP50: 75% (p < .0001). Increase in mean life span of *coq-3(ok506) −/−* fed GD1 compared to N2 fed OP50: 113% (p < .0001). Data were subjected to one-way ANOVA with Fisher’s test at a significance level of p < 0.05.

A growing body of evidence indicates that the increased life span of *C*. *elegans* fed the GD1 diet is not due to the lack of Q per se. *C*. *elegans clk1* mutants also show enhanced life span in response to the GD1 diet [17]. The *clk1* mutants lack Q but continue to produce rhodoquinone, an amino-isoprenylated quinone involved in anaerobic respiratory metabolism, as well as demethoxy-Q, the penultimate intermediate in Q biosynthesis [23,24]. To determine whether the GD1 diet would also act to extend life span of a *C*. *elegans* mutant with an earlier defect in the Q biosynthetic pathway, we tested the effects of this diet on two *C*. *elegans coq3* mutants. COQ-3 is an *O*-methyltransferase required for two steps of Q biosynthesis: the first *O*-methylation step precedes formation of the quinone ring, and the second *O*-methylation step is the final step, producing Q [25]. *C*. *elegans coq3* mutants have more severe phenotypes than the *clk1* mutants [20,26]. The *coq3* mutant worms respond to the GD1 *E*. *coli* diet when maintained on the diet either from time of hatching (Figure 2A), or when the diet is provided to the mutants upon reaching the L4 larval stage (Figure 2B). These results indicate that the GD1 diet imparts life span extension even to worm mutants with severe early defects in Q biosynthesis, and hence its effects are independent of worm Q content.

### Worms fed a mixture of GD1 and rescued GD1 show an intermediate life span extension

**Figure 3:**
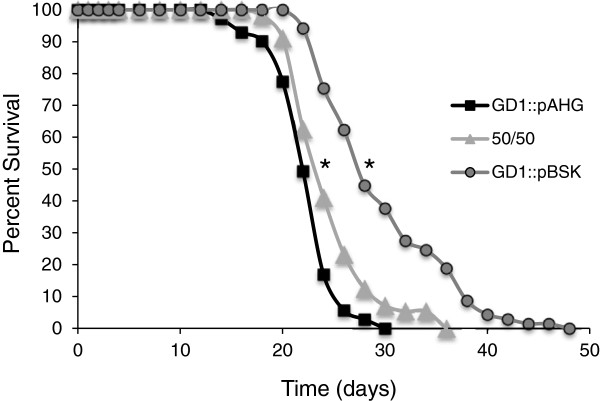
**Feeding worms GD1 in combination with rescued GD1 leads to improved survival compared to worms fed only rescued GD1.** L4 wild-type N2 worms were placed on NGM plates containing 12 μg/mL tetracycline and seeded with either GD1:pBSK cells only (circles, dark grey, n =71), GD1:pAHG cells only (squares, black, n = 69) or an equal mix of both cell types (triangles, light grey, n = 58). *Asterisks* designate: A significant increase in mean life span of worms fed GD1:pBSK compared to worms fed GD1:pAHG: 30% (p < .0001); Increase in mean life span of animals fed the mixed diet compared to GD1:pAHG alone: 9% (p < .0001). Data were subjected to one-way ANOVA with Fisher’s test at a significance level of p < 0.05.

It seemed likely that the life-span extension of GD1-fed animals might arise for two distinct reasons: (1) the GD1-diet mediated life span extension could be due to the presence of a beneficial component present in respiratory defective *E*. *coli*; or (2) to the absence of a toxic component present in respiratory competent *E*. *coli*. In order to distinguish between these two possibilities, we carried out a mixing experiment. Nematodes were fed the GD1:pBSK (respiratory deficient) diet, the rescued GD1 diet (GD1:pAHG, containing the wild-type *E*.*coli ubiG*), or a 50:50 mix. In order to prevent growth of the respiring cells from dominating the mixed diet, the *E*. *coli* were placed on NGM plates containing the bacteriostatic antibiotic tetracycline. Previous studies have shown that the GD1 mediated life span extension remains effective even when antibiotics inhibited bacterial proliferation [18]. Worms fed this *E*. *coli* mixture showed an intermediate degree of life span extension (Figure 3, Table 1). Although this result does not unambiguously identify one diet as beneficial or detrimental, it does indicate that the benefit of the GD1 diet takes effect even in the presence of respiratory-competent *E*. *coli*. However, the benefit of the mixed diet may depend on the presence of the bacteriostatic antibiotic.

**Table 1:** Statistical analyses of life spans

**Strain, food, treatment**	**n**	**mean** **±** **s.d. (dy)**	**max (dy)**	**% change in mean life span from control**	**p-value**
**N2, OP50**^**a**^	79	15 ± 4	20		
N2, GD1^a^	61	31 ± 5	38	+ 107	<.0001
**N2, OP50**^**b**^**(Adult)**	164	18 ± 3	29		
N2, GD1^b^	135	30 ± 5	34	+ 67	<.0001
*skn-1(zu169)−/−*, OP50^b^	153	16 ± 3	20	− 11	<.0001
*skn-1(zu169)−/−*, GD1^b^	131	27 ± 6	35	+ 50	<.0001
**N2, GD1::pAHG, – UV**^**c**^	52	18 ± 4	22		
N2, GD1::pBSK,–UV^c^	60	16 ± 4	22	− 11	.0001
N2, GD1::pAHG, + UV^c^	64	20 ± 3	22	+ 11	<.0001
N2, GD1::pBSK, + UV^c^	64	21 ± 3	23	+ 17	<.0001
**N2, GD1::pAHG only**^**d**^	71	23 ± 3	26		
N2, GD1::pBSK only^d^	69	30 ± 6	42	+ 30	<.0001
N2, Mixed^d^	58	25 ± 4	33	+ 9	<.0001
**N2, OP50**^**e**^	529	19 ± 5	27		
N2, GD1^e^	225	26 ± 8	39	+ 37	<.0001
*coq-3(ok506)−/−,* OP50^e^	119	15 ± 6	29	− 21	<.0001
*coq-3(ok506)−/−,* GD1^e^	102	30 ± 12	50	+ 58	<.0001
*coq-3(qm188)−/−,* OP50^e^	259	16 ± 5	25	− 16	<.0001
*coq-3(qm188)−/−,* GD1^e^	141	33 ± 18	63	+ 74	<.0001
**N2, OP50**^**f**^**(Adult)**	63	16 ± 4	22		
N2, GD1^f^	55	28 ± 7	40	+ 75	<.0001
*coq-3(ok506)−/−,* OP50^f^	84	8 ± 3	14	− 50	<.0001
*coq-3(ok506)−/−,* GD1^f^	53	34 ± 8	47	+ 113	<.0001

^a, b, c, d, e, f,^ identify cohorts from the same experiment. Within each cohort data were subjected to One-Way ANOVA analyses with Fisher's test at a significance of 0.05.

(p-values are compared to the condition in bold text for a given cohort).

### Worms fed GD1 are more thermotolerant and resistant to juglone treatment

**Figure 4:**
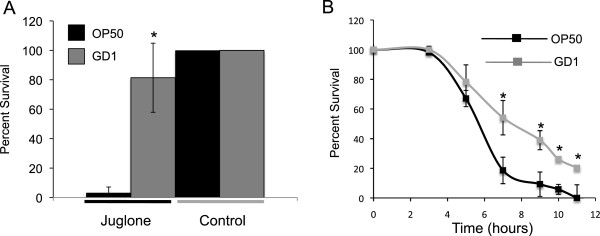
**GD1-fed worms are more resistant to juglone treatment and show enhanced thermotolerance.** (**A**) Wild-type N2 worms were fed OP50 or GD1 from the hatchling stage. L4 larval worms were placed in a drop of S-media containing either 250 μM juglone or an equal amount of ethanol vehicle control for 20 min. Worms were washed onto OP50 plates to recover and assayed for survival 18 h later. Black bar: OP50, grey bar: GD1; *Asterisk* indicates p-value = 0.0003 determined with Student’s t-test when compared to the OP50 + juglone condition. (**B**) Wild-type N2 worms were fed OP50 or GD1 from the hatchling stage. L4 larval worms were incubated at 35°C and survival was assessed at each indicated time point. Black line: OP50, grey line: GD1. *Asterisks* indicate p-values determined with Student’s t-test for comparisons between GD1 and OP50 at the designated time points: (7 h) 0.003; (9 h) 0.0013; (10 h) 0.0001; (11 h) 0.017.

Mutants of *C*. *elegans* with life span extension often show enhanced resistance to thermal and oxidative stress [10], suggesting that worms fed the GD1 diet would also demonstrate stress resistance. Juglone is a quinone that imposes both oxidative and electrophilic stress [27,28]. Juglone penetrates the worm cuticle and has been used to select for oxidative stress-resistant mutants [29]. As shown in Figure 4A, worms fed GD1 from the hatchling stage display improved survival following exposure to 250 μM juglone, as compared to similarly treated worms fed OP50. It is unlikely that the improved worm survival is due to hypersensitivity of GD1 *E*. *coli* to juglone treatment because the GD1 *E*. *coli* were actually more resistant to juglone treatment than OP50 *E*. *coli* (Additional file 1). Similarly, worms fed GD1 are more thermotolerant at the L4 stage compared to worms fed OP50 (Figure 4B).

### Excreted components present in GD1 *E*. *coli* spent media are not responsible for life span extension

**Figure 5:**
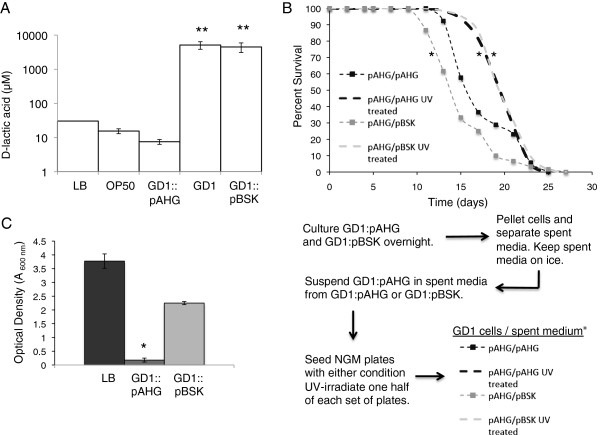
**Spent media from coenzyme Q-deficient *E. coli *contain high concentrations of D-lactic acid that serves as an energy source for respiring bacteria but has no direct effect on worm survival.** (**A**) GD1 *E. coli* has fermentative metabolism at normal oxygen levels. Spent media of indicated cultures or LB medium were assayed for D-lactic acid. *Asterisks* indicate p-values < 0.05 when compared to D-lactic acid content in OP50 spent media. (**B**) GD1:pAHG cells carrying a wild-type copy of the *ubiG* in a plasmid were suspended in either their own spent media or the respiration deficient GD1:pBSK spent media (see flow chart below panel **B**). One cohort of plates was UV-irradiated to kill the *E. coli* cells. Wild-type worms were fed these diets starting at the L4 larval stage. Diets were composed of *E. coli* cells suspended in: GD1:pAHG spent media (black squares, n = 52); GD1:pBSK spent media (grey squares, n = 60); GD1:pBSK spent media + UV (grey dashed line, n = 64); GD1:pAHG spent media + UV (black dashed line, n = 64). UV treatment of *E. coli* cells suspended in spent media increased nematode mean life span as compared to nematodes fed designated diets without UV treatment (p-value < .0001). For (**A** and **B**) data were subjected to one-way ANOVA with Fisher’s test at a significance level of p < 0.05. (**C**) *E. coli* cells from overnight GD1:pAHG cultures were pelleted and the spent media kept on ice. The cells were diluted to an A600nm of 0.1 in either LB medium (black), GD1:pAHG spent medium (dark grey), or GD1:pBSK spent media (light grey). Cultures were grown at 37°C, 250 rpm, and the A600nm was determined after 23 h. *Asterisk* indicates p-value < 0.05 determined with Student’s t-test for comparison of GD1:pAHG with GD1:pBSK.

Previous studies have shown that *E*. *coli* mutants with defects in the *ubiA* gene, required for Q biosynthesis, excrete large amounts of D-lactic acid in the spent media [30]. We found that the spent media of both GD1 and GD1:pBSK *E*. *coli* contain millimolar quantities of D-lactic acid (Figure 5A). In contrast, the spent media collected from cultures of OP50 contain only 10–20 μM D-lactic acid, similar to the concentration observed in LB media alone. Similarly, rescued GD1 cells containing a wild-type copy of *ubiG* produce very low levels of D-lactic acid, indicating that excretion of D-lactic acid by the GD1 *E*. *coli* is due to the loss of Q biosynthesis. The accumulation of lactic acid, in conjunction with the observation that *ubiG* mutant *E*. *coli* produce acetate under similar conditions [31], indicates that loss of Q forces the cells into a constitutive fermentative metabolic state despite the availability of oxygen.

To determine if the excreted D-lactic acid (or other fermentation products) present in GD1 spent media is responsible for the increased life span in worms fed this diet, we performed media swap experiments. Actively respiring rescued GD1 cells containing the *ubiG* gene on a plasmid (GD1:pAHG) were suspended in either their own spent media or the spent media from non-rescued GD1 cells (GD1:pBSK). Surprisingly, worms fed the GD1:pAHG cells suspended in the D-lactic acid rich spent media from GD1 cells, lived shorter lives than worms fed GD1:pAHG cells suspended in their own spent media (Figure 5B, Table 1). A separate cohort of each plate type was subjected to UV-treatment in order to prevent cells from metabolizing the D-lactic acid in the spent media. As shown in Figure 5B, worms do not display a difference in survival when fed UV-treated GD1:pAHG cells suspended in either type of spent medium. Both results indicate that the excreted components present in GD1 *E*. *coli* spent media are not responsible for life span extension.

Bacterial proliferation in the gut has been implicated as a major contributor of mortality in the worm [14,32]. We speculated that the respiring cells suspended in spent media containing large amounts of D-lactic acid were converting this fermentative product into energy-rich metabolites, fueling proliferation and other cellular functions. To test whether the D-lactic acid in the spent media does supply fuel for growth, we suspended overnight cultures of GD1:pAHG cells in either their own spent media, LB media or the spent media from GD1 cells. We found that the cells provided the GD1 spent media grew nearly as well as cells in LB media, whereas cells suspended in their own spent media showed negligible growth (Figure 5C). These results suggested that respiring *E*. *coli* cells utilize D-lactic acid and other metabolites present in the spent media as fuel for proliferation. Under these conditions, the utilization of D-lactic acid has a negative impact on worm life span (Figure 5B).

### Q deficient *E*. *coli* replicate more slowly than wild-type or ATP synthase mutant *E*. *coli*

**Figure 6:**
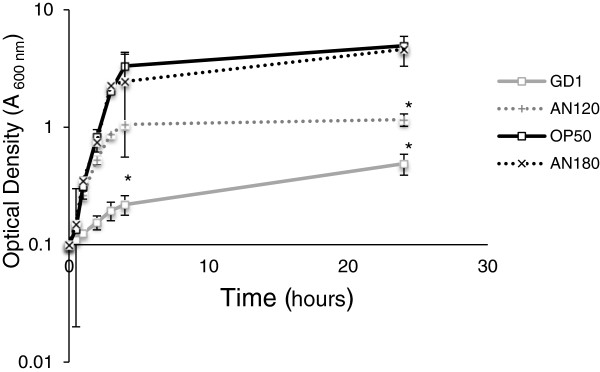
**GD1 *E. coli *proliferate more slowly than either wild-type or ATP synthase mutant *E. coli. *** Overnight cultures of the indicated *E. coli* strains were adjusted to an optical density (A_600 nm_) of 0.1 in LB medium containing the appropriate antibiotic. The increase in cell number was assayed over time. Solid grey line with open squares, GD1; dotted grey line with +, AN120 (ATP synthase mutant); solid black line with open squares, OP50; dotted black line with X, AN180 (wild-type parental strain of AN120). *Asterisks* indicate p-value < 0.05 when compared with A_600nm_ of OP50 culture at the 5 and 25 h time points. Data were subjected to one-way ANOVA with Fisher’s test at a significance level of p < 0.05 at each time point indicated.

Bacteria use a large proportion of available energy for replication; the loss of Q should lead to slow proliferation compared to wild-type cells. Bacterial proliferation inside the worm is known to influence life span [14]. The ATP synthase mutant strain AN120 has wild-type Q levels but is incapable of utilizing the proton-motive force to produce ATP [33]. The life span extension in worms fed AN120 is similar to that of worms fed an *E*. *coli* mutant (1100Δbc) harboring a deletion of the entire operon encoding ATP synthase [18]. Worms fed the *E*. *coli* parental strain 1100 had life spans indistinguishable from either OP50 or AN180 (the parent strain of AN120) [18]. Life spans of N2 worms fed rescued GD1 (GD1:pAHG) or OP50 are also indistinguishable [17]. Thus we determined the growth dynamics of representative bacterial strains known to influence life span. GD1 *E*. *coli* grow more slowly as compared to either OP50 or AN180 and also reach saturation at lower cell density (Figure 6). The AN120 mutant cells show an intermediate rate of growth and cell density at saturation (Figure 6). The bacterial proliferation observed is consistent with the hypothesis that worms fed diets of the slower growing *E*. *coli* strains have longer life span.

### *E*. *coli* deficient in respiration show lower colonization of the worm gut during early- to mid-adulthood

**Figure 7:**
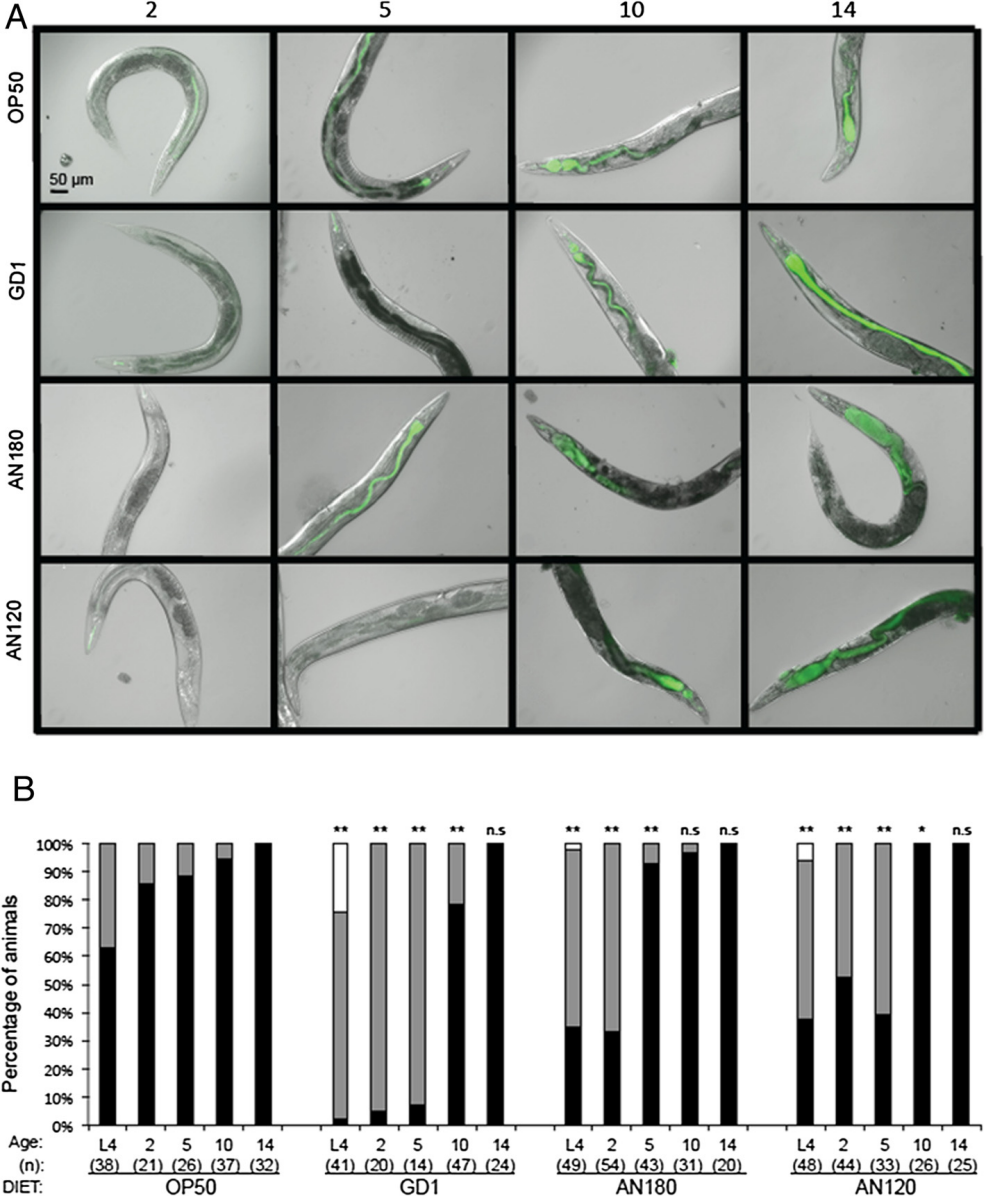
**Worms fed diets of GD1 or AN120 *E. coli *have decreased amounts of gut colonization as compared to worms fed OP50 or AN180 *E. coli. *** (**A**) Worms were fed OP50, AN180, GD1, or AN120 *E. coli* strains carrying a GFP-expressing plasmid from the hatchling stage and imaged at day two, five, ten and fourteen of adulthood. Images taken at days two and five were at 100 ms exposure, and images taken at days ten and fourteen at 50 ms exposure. (**B**) The percent of animals showing the absence (white bar) or presence of GFP-carrying *E. coli* in either the pharynx only (grey bar), or in both the gut and the pharynx (black bar), was determined at the indicated times. There were no animals with fluorescence in the gut only. The number of total animals scored (n) is indicated in parentheses. Data were subjected to Chi-squared analysis, with pairwise comparisons. *Asterisks* indicate *p-value < 0.05 or **p-value < 0.0001 as compared with age-matched OP50-fed worms; n.s., not significant. Pairwise comparisons were also performed for each of the ages sampled across the different diets (Additional file 4).

OP50 *E*. *coli* have been previously shown to colonize and proliferate in the worm gut [15,32]. Bacterial proliferation in the gut is considered a major contributor to worm mortality [14,32]. Similarly, we found that two day-old adult worms fed OP50 *E*. *coli* expressing GFP accumulate bacteria as evidenced by the green fluorescence throughout the gut (Figure 7A and B). This accumulation becomes more pronounced at day 5, and clusters of bacteria form distensions along the intestine. In contrast, worms fed GD1 expressing GFP do not show evidence of bacteria in their intestinal tracts at day 2 or 5. In fact, the few GFP-expressing bacteria evident in these animals reside only in the anterior part of the pharynx (Figure 7A and B, and Additional file 2). The apparent lack of passage through the pharynx into the intestine is not influenced by the size of the GD1 *E*. *coli*, because this strain is indistinguishable from OP50 in terms of cell size and shape (Additional file 3).

At day 5 of adulthood, worms fed the ATP synthase deficient *E*. *coli* AN120 strain display an intermediate degree of colonization of the intestine as compared to either OP50-fed worms or the AN180 parental strain (Figure 7B). Interestingly, worms fed AN180 displayed a diminished gut infiltration pattern as compared to OP50 at day two of worm adulthood (Figure 7A and B), despite growing to a thicker density on plates (data not shown). In contrast, from day five of adulthood onward, worms fed AN180 have intestinal GFP patterns identical to OP50-fed worms, indicating that the lag of AN180 infiltration occurs only during the early stage of worm adulthood (Figure 7A and B).

Worms fed OP50 to the tenth day of adulthood have distended intestinal lumens filled with GFP-labeled bacteria. Severe anatomical alterations of the gut deviating from the organ’s previously linear shape are prevalent (Figure 7A). Nonetheless, the degree of bacterial infiltration of the gut increased only slightly compared to day five animals (Figure 7B). By day 10, GD1-fed worms show appreciable amounts of gut bacteria-GFP fluorescence, yet the intestine is still not noticeably distended (Figures 7A and B). In contrast, 10 day-old worms fed AN120 accumulate gut bacteria-GFP fluorescence and acquire the distended gut appearance of worms fed OP50 (Figure 7A and B, and Additional file 4). By day 14 of adulthood all worms have large portions of the gut distended due to bacterial accumulation, regardless of the diet (Figure 7A). Every animal assayed at day 14 demonstrates intestinal accumulation of *E*. *coli* (Figure 7B). These results suggest that early accumulation of bacteria in the nematode gut is linked to a shorter nematode life span.

### Worms fed GD1 have decreased coliform counts

**Figure 8:**
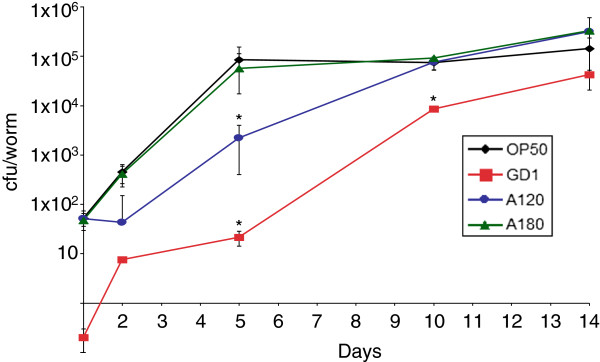
**Worms fed respiratory deficient *E. coli *have decreased coliform counts during early to mid adulthood.** N2 worms were fed OP50, AN180, GD1 or AN120 as hatchlings and five worms were collected and mechanically disrupted at the designated age of adulthood. The lysate was analyzed for colony forming units as described in Experimental Procedures. Colony forming units (cfu/worm) were determined the following day. (Note that N2 L4 larvae contained on average less than 1 cfu/worm). Black diamonds, OP50; red squares, GD1; green triangles, AN180; blue circles, AN120. *Asterisks* indicate p-value < 0.05 when compared with the OP50 diet on the designated day. Data were subjected to one-way ANOVA with Fisher’s test at a significance level of p < 0.05 for each time point indicated.

These findings indicated that the worms accumulated bacteria in their intestine to different extents depending on their diet. However, this assay was qualitative in nature. To quantify the colony density within the intestinal lumen of individual animals, worm lysates were prepared from animals fed either the OP50 or GD1 diets from time of hatching. The worms were collected at various ages ranging from the L4 larval stage to day 14 of adulthood and the number of colony-forming units retrieved per worm (cfu per worm or coliform counts) determined. The coliform counts varied dramatically between GD1 and OP50-fed animals. We measured an average of 10 cfu/worm in GD1-fed day five adult worms as compared to 1 × 10^5^ cfu/worm in age-matched worms fed either OP50 or AN180 (Figure 8). Worms fed OP50 reached a saturation point by day five, whereas worms fed GD1 showed a linear progression of coliform counts, but did not reach OP50 counts even by day 14.

Interestingly, the cfu/worm in *C*. *elegans* fed AN120 were intermediate as compared to OP50, AN180, or GD1, particularly at days 2 and 5 of adulthood (Figure 8). However, day 10 and 14 adult animals fed AN120 have similar coliform counts as compared to either OP50 or AN180 fed worms (Figure 8).

## Discussion

The life span of *C*. *elegans* fed diets of respiratory deficient *E*. *coli* is significantly enhanced as compared to *C*. *elegans* fed the standard lab diet of OP50 *E*. *coli* (Figures 1 and 2, Table 1) and [17,18]. These benefits are not confined to long-term survival, because animals fed the GD1 bacterial strain fare better than worms fed OP50 during short-term stress assays such as exposure to the oxidative agent juglone or to high-temperature (Figure 4). The *E*. *coli* respiratory deficiency, due to either the lack of Q or a deficiency in complex V, mediates worm life span extension and increased stress resistance independent of dietary restriction or the worm Q content. Worms fed the standard OP50 *E*. *coli* diet have distended guts packed with *E*. *coli* and show maximal coliform counts (cfu/worm) by day five of adulthood. However, worms fed the Q-less GD1 *E*. *coli* show delayed gut colonization and coliform counts fail to reach maximal levels even by day 14. The findings reported here suggest that the delayed replication of respiratory deficient *E*. *coli* in the gut lumen confers a survival benefit to the animal that correlates with the longer worm life span and enhanced stress resistance.

A recent study has suggested that the degree of bacterial colonization of the intestine at day two of *C*. *elegans* adulthood can be utilized as a predictor of subsequent worm survival 6 – 24 days thereafter [32]. We have found that this predictive window can be extended to the fifth day of adulthood. It has been previously shown that worms fed OP50 or AN180 have similar life spans [18]. Coliform counts (cfu/worm) in animals fed these diets are similar (Figure 8) when assayed at the L4 larval stage and throughout adulthood. In contrast, worms fed the ATP synthase defective *E*. *coli* strain AN120 yield coliform counts intermediate to OP50 and GD1 until day ten, when the values become similar to those of OP50-fed animals (Figure 8). Similarly, coliform counts from GD1-fed worms are significantly lower than worms fed any of the other diets at day two, five, or ten of adulthood (Figure 8). These findings suggest that the coliform counts at days two and five are predictive of the enhanced life span in worms fed these diets.

What accounts for the dramatically low coliform counts in the GD1-fed animals? It seems likely that the pharynx, which is responsible for grinding the food taken up by the worm, efficiently breaks down the Q-deficient *E*. *coli*. This degradation could exert an “abiotic” condition in the guts of animals fed this diet. Subsequently, GD1-fed worms begin accumulating bacteria in their guts by day ten of adulthood (Figures 7A, 7B, and 8). The transition from mid to late adulthood marks a shift in pharyngeal function [13,14]. Animals become plagued by the effects of sarcopenia, or muscle wasting, as they age [12]. The pharynx muscle declines in pumping activity and shows increasing tissue disorder [13,14]. Indeed, the pharyngeal pumping rate of worms significantly diminishes between early and mid-adulthood [13]. The intestinal colonization patterns of *phm2* mutant worms, which have a poorly functioning pharynx, indicate that large amounts of bacteria pass through the grinder intact. These animals have life spans considerably shorter than wild-type worms, and concomitantly higher numbers of *E*. *coli* in the gut lumen [32]. Interestingly, bacteria are considered to play only a minor role in the decline of this organ, implying that degeneration of the pharynx was due predominantly to the effects of long-term pumping [13]. Feeding *C*. *elegans* pathogenic bacteria such as *Salmonella* or *Serratia marcescens* degrades performance of the pharyngeal grinder and allows early passage of bacterial cells to the worm intestine [34,35]. Our results suggest that the type of *E*. *coli* diet can profoundly alter the “functional aging” of the pharynx. We speculate that the Q-less *E*. *coli* membranes may be especially fragile when subjected to the worm pharyngeal grinder due to the absence of Q, which normally serves to maintain membrane stability by acting as a crucial membrane chain-terminating antioxidant [36]. Taken together, these findings underscore the importance of efficient bacterial degradation, as the number of intact bacteria that make it past the pharyngeal grinder clearly impact worm survival.

Replicating bacteria in the gut have already been implicated as a main contributor of worm death [14]. Worms fed either UV-irradiated or antibiotic-treated OP50 had increased survival [14,18,37]. Similarly, *C*. *elegans* exposed to UV-irradiated *Enterica faecalis* or *Salmonella* displayed greater survival than animals fed viable cells of these pathogenic strains [38,39]. However, worms fed UV-irradiated GD1 *E*. *coli* exhibited shorter life span than worms fed untreated GD1 [18]. We have observed enhanced susceptibility of GD1 *E*. *coli* to UV treatment. We speculate that the UV-treatment of GD1 as performed previously [18] actually represents a vast overdose of that required for cell killing, and may result in a toxic food that fails to support larval development (data not shown). Alternatively, it is possible that worms recognize metabolites produced by GD1 cells, similarly to those produced by OP50, and respond through up-regulation of antimicrobial genes. Thus, GD1 cells that are able to reside within the gut lumen may act to elicit different worm signaling pathways that control innate immunity and the expression of antimicrobial genes such as *lys8*[40]. In our study, the delay in *E*. *coli* accumulation of the gut in worms fed GD1 confers a survival advantage in the animal, and it will be important to determine whether the GD1 diet-mediated longevity effects can be attributed to enhanced intestinal immunity through known signaling pathways [32]. The diminished proliferative capacity of the Q-deficient *E*. *coli* (Figure 6) serves to retard the level of infection in the gut once intact bacterial cells settle within the lumen of the aged worm. Therefore, we propose that both Q and ATP synthase function be considered virulence factors.

Both Q and ATP synthase serve essential functions in respiratory metabolism. A growing body of evidence suggests that bacterial pathogens within the gastrointestinal tract must sense oxygen availability (or lack thereof) and their metabolic adaptation to the host environment plays a key role in the expression of virulence factors and in modulating host responses [41]. In *E*. *coli* ArcB senses oxygen availability via the quinone redox status (Q/QH_2_ and menaquinone/menaquinol) and tunes aerobic and anaerobic respiratory metabolism through its phosphorylation of ArcA [42]. ArcA functions as a transcriptional regulator of operons involved in respiratory and fermentative metabolism; ArcA plays a role in virulence in a wide variety of pathogenic bacteria in animals and humans including the enteric pathogens *Vibrio cholerae*[43] and *Shigella flexneri*[44]. Mutations in genes encoding respiratory chain complexes also identify components in pathogens essential for virulence. Rat lung fibroblasts exposed to *Shigella flexneri* with mutations in the cytochrome *bd* oxidase had lower numbers of plaques than fibroblasts infected with the wild-type parental strain [45]. *Brucella abortus*, a zoological pathogen that causes spontaneous abortions in cattle, showed attenuated virulence against murine macrophages after the cytochrome *bd* oxidase gene was disrupted [46]. Two examples directly underscore the relationship between respiration, proliferation and pathogenicity. *Burkholderia cenocepacia* mutants lacking a functional phenylacetic acid catabolism pathway, which degrades aromatic compounds and shunts electrons into the TCA cycle, grow slowly and are less virulent to *C*. *elegans* than wild-type *B*. *cenocepacia*[47]. Bae and colleagues fed *C*. *elegans* mutated *Staphylococcus aureus* generated in a random disruption screen and found that disruption mutants in various TCA cycle genes showed attenuated killing activity [48]. Taken together, the findings presented here and in other model systems identify respiration and energy production as important virulence factors.

Our findings indicate that excreted components present in GD1 *E*. *coli* spent media are not responsible for worm life span extension. GD1 excreted large amounts of D-lactic acid into its media during growth (Figure 5A). The *E*. *coli ubiA* mutant, deficient in a different Q biosynthetic reaction, also accumulates large amounts of D-lactate under normoxic conditions [30]. Intriguingly, consumption of lactic acid is beneficial in a variety of organisms. Ikeda and colleagues showed that worms lived longer and were more resistant to *Salmonella enterica* infection when fed the D-lactic-acid producing bacteria *Bifidobacterium sp*. or *Lactobacillus sp*., although whether this was due to the lactic acid itself was not shown [16]. Feeding heat-killed species of *Lactobacilli* increased worm life span, implying that the lactate in these cultures was responsible for the improved survival [49]. However, these results are difficult to interpret because the authors compared killed *Lactobacilli* to living OP50. It is crucial to consider the stereoisomer of lactic acid provided during these analyses. *E*. *coli* produces D-lactic acid under hypoxic conditions [50], whereas *C*. *elegans* lactic acid dehydrogenase is considered specific for the L-stereoisomer [51]. Thus, the worm is incapable of converting the D-lactic acid produced by the bacteria into pyruvate. These considerations clarify the results of the spent media/mixing experiment, because while worms cannot utilize the D-lactic acid present in the spent medium of GD1 cultures, rescued GD1 *E*. *coli* are able to utilize the D-lactic acid (Figure 5B and 5C). For this reason, the D-lactic acid present in the spent media had no effect on *C*. *elegans* life span unless it was provided in combination with respiratory competent *E*. *coli*, in which case it led to more bacterial proliferation and a shorter worm life span.

It is becoming clear that certain pathological and aging-related disorders are related to the composition of the intestinal microflora [1]. The use of beneficial bacteria to influence the health status of humans is quickly becoming a viable therapeutic option. Premature infants given *Lactobacilli* soon after birth show significantly decreased incidents of necrotizing enterocolitis [52]. Probiotic therapies have an anti-cancer effect in human patients [53], while changes in intestinal microbiota composition were associated with the decreased onset of intestinal tumors in the cancer prone ApcMin mouse strain [2]. Mice fed *Bifidobacterium animalis* subspecies lactis lived longer than littermates fed a control diet and showed diminished gut inflammation [9]. Fruit flies require certain bacteria in their guts for healthy metabolism [54]. Probiotic interventions have yielded promising results in worms [16]. A recent study showed that the folate status of the gut microbiome may slow *C*. *elegans* aging [55]. In the presence of tetracycline, the worms assayed in our study responded well to a mixed diet composed of Q-replete and Q-deficient *E*. *coli* (Figure 2), indicating that the benefit of the GD1 diet takes effect even in the presence of respiratory-competent *E*. *coli*.

In summary, our study argues that *E*. *coli* respiration is a virulence factor of OP50 *E*. *coli*, the standard lab diet of *C*. *elegans*. The decreased coliform counts present in worms fed respiratory deficient *E*. *coli* may manifest in at least two ways: (1) the lack of Q increases the tendency of the pharyngeal grinder to break apart the *E*. *coli* GD1 cells; (2) the respiratory deficiency of both the Q-less and ATP synthase mutants may render them less able to colonize the gut once the intact bacteria have infiltrated. The delayed accumulation and diminished presence of bacteria in the gut of the animal attenuates the stress encountered by the aging animals, yielding longer-lived worms

## Conclusions

We show that the respiratory deficient *E*. *coli* diet imparts not only longer life span, but also increased resistance to thermal stress and juglone treatment. The longevity observed is independent of the worm Q content and dietary restriction. We provide evidence that the decreased accumulation of respiratory deficient bacteria in the worm intestine is responsible for the increased longevity observed in *C*. *elegans*. The lack of Q in particular makes the bacteria more susceptible to degradation at the worm’s pharynx. In summary, we put forward the idea that respiration is a virulence factor that has a profound effect on the ability of *E*. *coli* to colonize and harm its host.

## Methods

### *C*. *elegans* strain and growth conditions

*C*. *elegans* strains are listed in Table 2. *C*. *elegans* were maintained under standard conditions at 20°C unless otherwise indicated [56]. Wild-type (N2, Bristol) and the EU35 *skn1*(*zu169*) mutant were acquired from the Caenorhabditis Genetics Center (Minneapolis, MN). The *coq3* mutants CFC1005 and CFC315 were previously described [20]. Nematode growth medium was prepared as previously described unless stated otherwise [56].

**Table 2:** ***C. elegans *and *E. coli *strains used in this study**

**Strain**	**Genotype**	**Source**
***C. elegans***		
N2	wild-type	CGC
EU35	*skn-1(zu169)* IV/nT1 [unc?*(n754*) let?] *(IV;V)*	CGC
CFC1005	*coq-3(qm188)/*nT1[qIs51]	[20]
CFC315	*coq-3(ok506*)/nT1[qIs51]	[20]
***E. coli***		
OP50-1		CGC
GD1	*ubiG*::*Kan*, *zei::Tn10dTet*	[57]
GD1:pBSK	*ubiG*::*Kan*, *zei::Tn10dTet*:pBSK	this report
GD1:pAHG	*ubiG*::*Kan*, *zei::Tn10dTet*:*ubiG*	[57]
AN120	*argE3, thi-1, str*^*R*^*, uncA401*	[33]
AN180	*argE3, thi-1, str*^*R*^	[33]
OP50-1:pFVP25.1		CGC
GD1:pFVP25.1		this report
AN120:pFVP25.1		this report
AN180:pFVP25.1		this report

### Growth of *E*. *coli*

Nematode diets consisted of *E*. *coli* strains listed in Table 2. *E*. *coli* were cultured in LB medium with the designated antibiotic and incubated overnight at 37°C with shaking at 250 rpm. *E*. *coli* cells were then harvested and seeded onto NGM plates containing the stated antibiotic. OP50-1 *E*. *coli* carrying an integrated streptomycin resistance gene (CGC) were cultured in the presence of streptomycin (250 μg/mL final concentration). GD1 *E*. *coli*, a Q-less strain harboring an insertion in the *ubiG* gene (*ubiG*::*Kan*, *zei*::*Tn10dTet*) [57], were cultured in the presence of kanamycin (100 μg/mL final concentration).

GD1:pAHG harbors a wild-type copy of the *E*. *coli ubiG* gene on a multicopy plasmid (pAHG) [57]. pBluescript (pBSK; Fermentas) was used as an empty vector control. Both GD1:pAHG and GD1:pBSK cells were grown overnight in LB media containing 100 μg/mL ampicillin.

The ATP synthase deficient *E*. *coli* strain AN120 and the parent strain AN180 were previously described [33]. Cultures of AN120 and AN180 were grown overnight in LB medium.

OP50 containing the pFVP25.1 plasmid with the GFP marker was acquired from the Caenorhabditis Genetics Center. GD1, AN180 and AN120 *E*. *coli* were also transformed with this plasmid. All *E*. *coli* strains carrying the pFVP25.1 plasmid were cultured in LB containing 100 μg/mL ampicillin and seeded to NGM plates containing 100 μg/mL ampicillin as described above.

### Determination of *C*. *elegans* total life span and adult life span

To determine *C*. *elegans* total life span (defined as the number of days from hatching until death), N2, CFC1005 and CFC315 gravid adults were hypochlorite lysed and eggs transferred to NGM plates containing the designated *E*. *coli* diet. Two days after hatching *coq*-*3* homozygous mutant worms were selected and transferred to plates containing the designated diet. N2 worms were similarly treated. A total of five or six plates per condition were used (20 worms per plate). Worms were scored for survival and moved to new plates every day for the first six days, then every four days thereafter while scoring for survival every two days. Worms that responded to being gently prodded with a platinum wire by moving or pharyngeal pumping were counted as alive. Worms with internally hatched larvae, an extruded vulva, or that escaped were censored from the total count. One-way ANOVA analyses of life spans were performed with StatView 5.0.1 (SAS, CA) software at a significance level of 0.05. Similar results were attained when data were subjected to Kaplan-Meier Test at a 0.05 significance level. Maximum life span was calculated from the mean of the top 10% longest lived worms, for each condition.

To determine *C*. *elegans* adult life span, N2, CFC315 and EU35 heterozygous gravid adults were hypochlorite lysed and eggs placed on NGM plates containing fresh OP50. After reaching the L4 larval stage, N2, *coq*-*3*(*ok506*) –/ – and *skn*-*1*(*zu169*) –/ – L4 larvae were transferred to separate plates containing either OP50 or GD1 *E*. *coli*, and the life span determined as described above.

### Media swap and UV-treatment of GD1:pAHG *E*. *coli*

GD1:pAHG and GD1:pBSK cells were grown as described above. The cells were pelleted, the spent media was removed and kept on ice, and the GD1:pBSK cells were discarded. An equal volume of GD1:pAHG cells were resuspended in either their own spent media or the spent media of the GD1:pBSK cells. These suspensions were then seeded onto regular NGM plates, allowed to dry at room temperature, and stored at 4°C until use. Half of the plates containing GD1:pAHG cells in GD1:pAHG spent media and half of the plates containing GD1:pAHG cells in GD1:pBSK spent media were UV-irradiated for 10 minutes at 365 nm on high setting with a Fluorchem2 imaging apparatus (Alpha Innotech, CA). N2 hatchlings were fed OP50 until the L4 larval stage, and then transferred to plates containing one of the designated diets: GD1:pAHG *E*. *coli* cells suspended in spent media obtained from cultures of either GD1:pAHG or GD1:pBSK; alternatively these two types of diets were first subjected to UV irradiation prior to the transfer of L4 larvae. Adult life span determinations were performed as described above.

### Preparation of mixed *E*. *coli* diets

The GD1:pAHG and GD1:pBSK cells were grown as described above and the optical densities (A_600nm_) were adjusted to 6.0. One cohort of each cell type was seeded onto NGM plates containing 12 μg/mL tetracycline. Another cohort of GD1:pAHG and GD1:pBSK at an optical density of 6.0 (A_600_) cells were combined at equal volumes, mixed well and seeded onto NGM plates containing 12 μg/mL tetracycline. Wild-type worms were hypochlorite lysed, transferred to NGM plates and fed OP50 as hatchlings. The L4 larvae were transferred as described above onto plates bearing one of three diets: GD1:pAHG cells only, GD1:pBSK cells only or an equal mix of GD1:pAHG and GD1:pBSK cells. Adult life span determinations were performed as described above.

### Measurement of D-lactic acid

OP50, GD1, GD1:pAHG and GD1:pBSK cells were grown overnight as described above. The cells were pelleted, the spent media was removed and saved on ice. Levels of D-lactic acid in the spent media were assayed using the Enzychrom D-lactate Assay Kit (BioAssay System Co., Hayward, CA), per the manufacturer’s instructions with an uQuant plate reader at 560 nm (Bio-Tec Instruments Inc., VT). The GD1 and GD1:pBSK spent media were diluted 1:10 with LB. One-way ANOVA analyses were performed with StatView 5.0.1 (SAS, CA) software at a significance level of 0.05, comparing all groups to D-lactic acid levels in OP50 spent media.

### *E*. *coli* growth determination

OP50:pFVP25.1, GD1:pFVP25.1, the ATP synthase deficient *E*. *coli* strain AN120:pFVP25.1 and its parent strain AN180:pFVP25.1 were grown overnight in LB media containing 100 μg/mL ampicillin. Optical densities were adjusted to 0.1 with LB media, and antibiotic was added for each strain. Bacteria were grown (37°C, 250 rpm) and the cell density was monitored over time by monitoring absorbance at 600 nm with a Shimadzu UV-160 spectrophotometer (Shimadzu, El Cajon, CA). One-way ANOVA analyses were performed with StatView 5.0.1 (SAS, CA) software at a significance level of 0.05, comparing optical density (A_600 nm_) of all groups versus OP50.

### *E*. *coli* growth determination in spent media

GD1:pAHG and GD1:pBSK cells were cultured overnight as described above. The cells were pelleted and the spent media saved on ice. The GD1:pAHG cells were diluted to an optical density of 0.1 in either LB media, spent media from GD1:pBSK cultures, or spent media from GD1:pAHG cultures. Absorbance (600 nm) was determined after 23 h of incubation. One-way ANOVA analyses were performed with StatView 5.0.1 (SAS, CA) software at a significance level of 0.05.

### Determination of *E*. *coli* cell size

OP50 and GD1 cells were grown as described above. Cells were placed onto glass slides and briefly heat fixed. The cells were DIC-imaged and photographed with a Deltavision Spectris Deconvolution Microscope system (Applied Precision). Linear measurements of cells were determined with the linear measurement tool. Fifteen cells per condition were measured. Student’s *t*-test at a significance level of 0.05 was used to analyze differences in size between the two strains.

### Thermal tolerance assay

Gravid wild-type worms were hypochlorite lysed and transferred to NGM plates containing either OP50 or GD1. Ten L4 larvae per plate (three plates were used for each condition) were subjected to 35°C heat stress and monitored for survival until all the worms on OP50-seeded plates were exterminated. Survival was assayed by gently prodding with a platinum wire. Dead worms were removed. The assay was conducted four times. Student’s *t*-test at each time point was used to assess differences of survival at a significance level of p < 0.05.

### Juglone survival assay

Gravid wild-type worms were hypochlorite lysed and eggs transferred to NGM plates containing either OP50 or GD1. Approximately 30 L4 worms were then placed in a 30 μL drop of S-media containing 250 μM juglone (Sigma) from a 12 mM stock solution in 100% ethanol. A drop of S-media containing an equal amount of alcohol was used as a vehicle control. The worms were maintained in the drop for 20 min and washed off the slide with 100 μL S-media onto NGM plates containing OP50. Worms were scored for survival 18 hours later.

For bacterial juglone survival assays, OP50 and GD1 were grown overnight in their respective media containing antibiotics. Cultures were diluted to 1.0 OD_600 nm_ in water, and resuspended in either 125 μM juglone or equal amounts of ethanol as vehicle control. The cells were incubated at 37°C with aeration (250 rpm) and at the indicated time points 3 μL aliquots were spotted onto LB plates containing the respective antibiotic in 1/10 dilutions. Plates were incubated at 37°C for 12 to 16 hours. The assay was conducted three times.

### Determination of coliform counts

Gravid wild-type worms were hypochlorite lysed onto NGM plates containing OP50:pFVP25.1, GD1:pFVP25.1, AN120:pFVP25.1 or AN180:pFVP25.1. The hatchlings were fed the designated diets and collected at the following stages: L4, two-, five-, ten-, or fourteen-days of adulthood. Five worms from each condition were washed in 5 μL of S-media with 0.1% Triton X-100 on a foodless NGM plate for 30 s. The worms were washed four times in total and then placed in a 0.5 mL microcentrifuge tube containing 20 μL of the S-media with 0.1% Triton X-100. Worms were mechanically disrupted with a micro-pestle (Sigma) for 200 strokes. The micro-pestle was placed in a 1.5 mL Eppendorf tube containing 100 μL S-media for 30 s, and the contents of the two tubes were combined. The contents of the tube were mixed well and spread onto plates containing 100 μg/mL ampicillin. Serial dilutions (1:1,000, 1:10,000 and 1:100,000) were prepared from worm lysates derived from the OP50- and AN180-diet conditions at the day two, five, ten, and fourteen adult time points. Serial dilutions (1:100, 1:1,000, and 1:10,000) were prepared from worm lysates derived from the GD1- and AN120-diet conditions at the day five, ten, and 14 adult time points. Colony forming units were determined the following day. In order to assay whether the micro-pestle mediated lysis of the worms affected the viability of the bacteria, an equal number of either OP50 or GD1 cells were subjected to mechanical disruption and the cfu quanitified in an identical fashion except that worms were omitted. The process of mechanical disruption did not affect the viability of either the OP50 or GD1 cells (data not shown). One-way ANOVA analyses were performed with StatView 5.0.1 (SAS, CA) software at a significance level of 0.05, comparing all conditions to OP50 fed worms at each indicated time point.

### Fluorescence microscopy and intestinal infiltration assay

To monitor bacterial proliferation within animals, synchronized N2 embryos were extracted from gravid adults following hypochlorite treatment and cultivated on OP50:pFVP25.1, GD1:pFVP25.1, AN120:pFVP25.1 or AN180:pFVP25.1 bacterial lawns on NGM plates containing 100 μg/mL ampicillin. Adult animals were moved to new plates every two days to prevent larval contamination. For imaging, L4 larvae and day two, five, ten, and fourteen adult nematodes were washed three times for 30 s in 30 μL M9, then placed onto slides prepared with fresh 2% agar pads. Worms were anesthetized with 100 mM levimasole (tetramisole hydrochloride, Sigma). GFP fluorescence in the pharyngeal or intestinal lumen was determined by visual inspection at 10X magnification on the Zeiss Imager M1 Axioscope. Fluorescent and Nomarski images were captured at 10X magnification using a Zeiss Axioimager A2 with an attached Zeiss AxioCam camera controlled by the software package Zeiss AxioVision.

The number of worms displaying bacterial fluorescence in the pharynx only, the gut only, or both the pharynx and gut were scored based on these images. These categories were chosen to assay the presence of the above-background fluorescence imparted by the bacteria carrying the GFP-expressing plasmid along the entire gastrointestinal tract; no distinction was made in the absolute levels of fluorescence in these categories. Representative mages were chosen to display the predominant category for each time point and diet. The results were pooled and subjected to Chi-squared analysis. The null hypothesis was ascertained as the values attained from OP50 fed animals.

### Statistical analyses

Student’s T-tests were used to determine significance of single comparisons. One-way ANOVA analyses with Fisher's test were performed with StatView 5.0.1 software (SAS, CA) at a significance level of 0.05 for all multiple comparisons. Chi-square tests were utilized in Figure 7B and Additional file 4.

## Abbreviations

CGC: Caenorhabditis Genetics Center; LB: Luria Broth; Q: Coenzyme Q.

## Competing interests

The authors declare that they have no competing interests.

## Authors’ contributions

FG and GM designed, planned, and conducted experiments, data/statistical analyses, data interpretation, and manuscript preparation. RS designed, planned, and conducted experiments, data/statistical analyses, and data interpretation. VT, EW, and LL conducted experiments. CS provided experimental design and data interpretation. CFC and AF provided scientific input, experimental design, data/statistical analyses, data interpretation, and manuscript preparation. All authors read and approve the final manuscript.

## Supplementary Material

Additional file 1 **OP50 are more sensitive to juglone than GD1. ***E*. *coli* cells were treated with either 125 uM juglone in ethanol, an equivalent volume of water, or an equivalent volume of ethanol for 2 h. Serial dilutions were prepared (undiluted, 1/10, 1/100, and 1/1000) and spotted onto LB + ampicillin plate medium. Pictures were taken after 24 and 48 h of incubation time at 37°C. Both strains carry a GFP plasmid (pFVP25.1). Click here for file

Additional file 2 **Close-up view of day five adult worms fed OP50 or GD1 *E*. *coli *diets.** Worms were fed OP50 or GD1 *E*. *coli* strains carrying a GFP-expressing plasmid from the hatchling stage and imaged at day five of adulthood. GFP-*E*. *coli* are evident as a large bolus in the anterior gut of the OP50-fed worm (left panel); GFP-*E*. *coli* are evident only in the anterior pharynx in the GD1-fed worm (right panel) (scale bar = 50 um). Click here for file

Additional file 3 **GD1 and OP50 *E*.*coli *are similar in size.** OP50 and GD1 E. coli cultures were grown overnight and visualized as described in Methods and Materials. Fifteen cells were measured per strain. The line traversing the cell in the OP50-panel demonstrates the dimension measured. Data subjected to Student’s t-test at a significance level of p < 0.05. Click here for file

Additional file 4 **Pairwise comparisons across diet and age.** The percent of animals showing the absence (white bar) or presence of GFP-carrying *E*. *coli* in either the pharynx only (grey bar), or in both the gut and the pharynx (black bar), was determined at the indicated times in Figure 7B. Asterisks indicate * p-value < 0.05 or ** p-value < 0.01 by pairwise Chi-square tests. Comparisons were performed for each of the ages sampled across the different diets. Click here for file
