# Supplementary material for: Delayed accumulation of intestinal coliform bacteria enhances life span and stress resistance in Caenorhabditis elegans fed respiratory deficient E. coli
Source: BMC Microbiol. 2012 Dec 20;12:300. doi: 10.1186/1471-2180-12-300 (PMC3548685; doi:10.1186/1471-2180-12-300)
Supplement: Additional file 1 — OP50 are more sensitive to juglone than GD1. E. coli cells were treated with either 125 uM juglone in ethanol, an equivalent volume of water, or an equivalent volume of ethanol for 2 h. Serial dilutions were prepared (undiluted, 1/10, 1/100, and 1/1000) and spotted onto LB + ampicillin plate medium. Pictures were taken after 24 and 48 h of incubation time at 37°C. Both strains carry a GFP plasmid (pFVP25.1). [file 1471-2180-12-300-S1.pptx]

## Slide 1
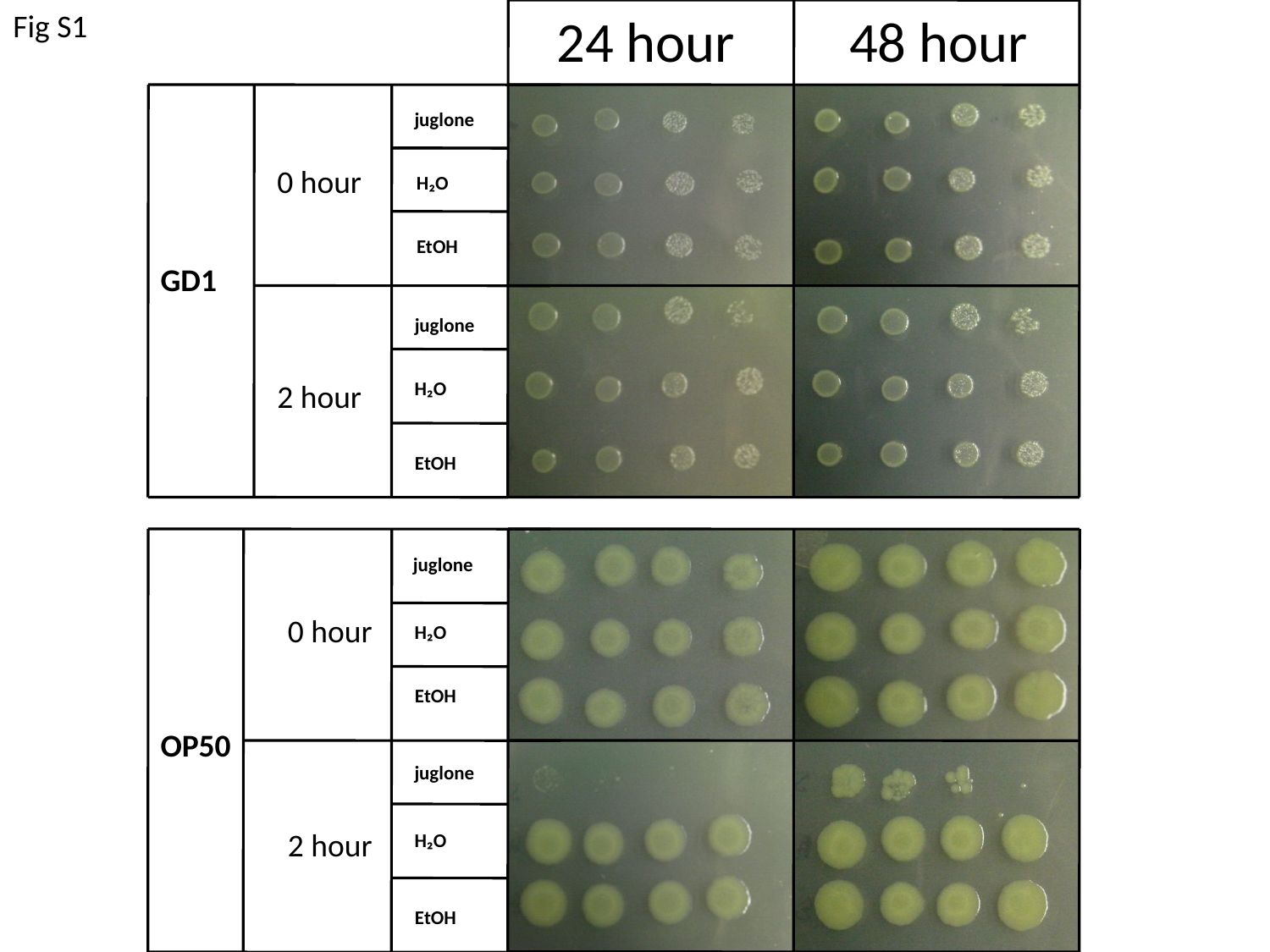

24 hour 48 hour
Fig S1
juglone
0 hour
H₂O
EtOH
GD1
juglone
2 hour
H₂O
EtOH
juglone
0 hour
H₂O
EtOH
OP50
juglone
2 hour
H₂O
EtOH
