# Supplementary figures and images for: Delayed accumulation of intestinal coliform bacteria enhances life span and stress resistance in Caenorhabditis elegans fed respiratory deficient E. coli
Source: BMC Microbiol. 2012 Dec 20;12:300. doi: 10.1186/1471-2180-12-300 (PMC3548685; doi:10.1186/1471-2180-12-300)

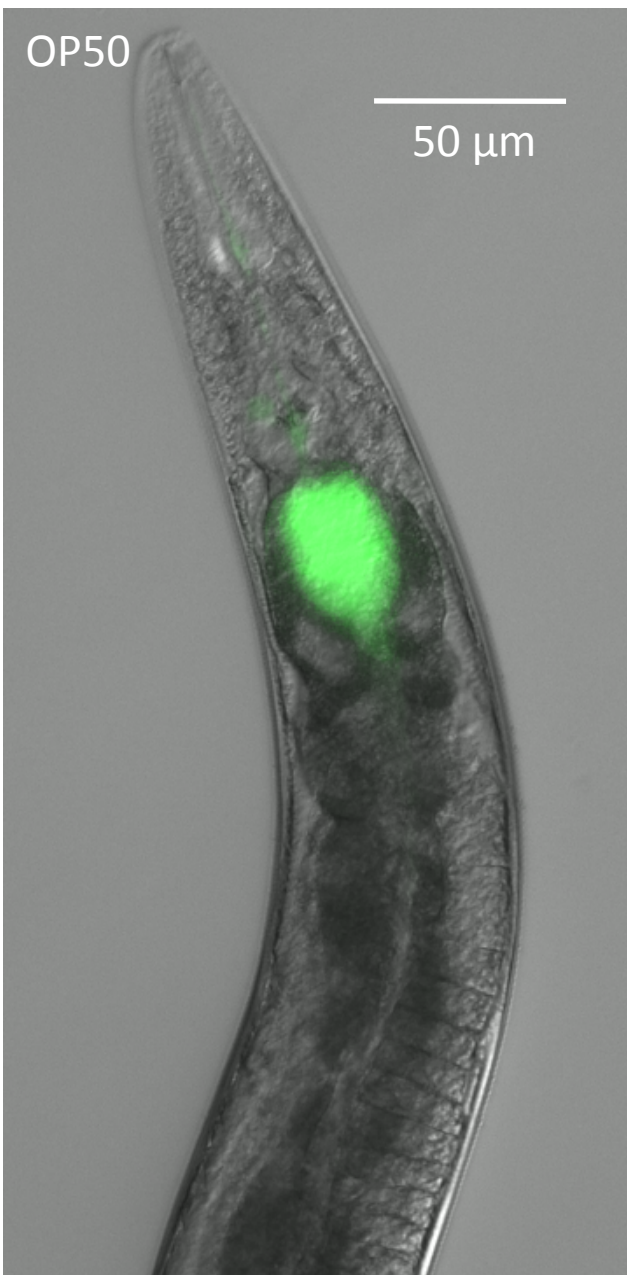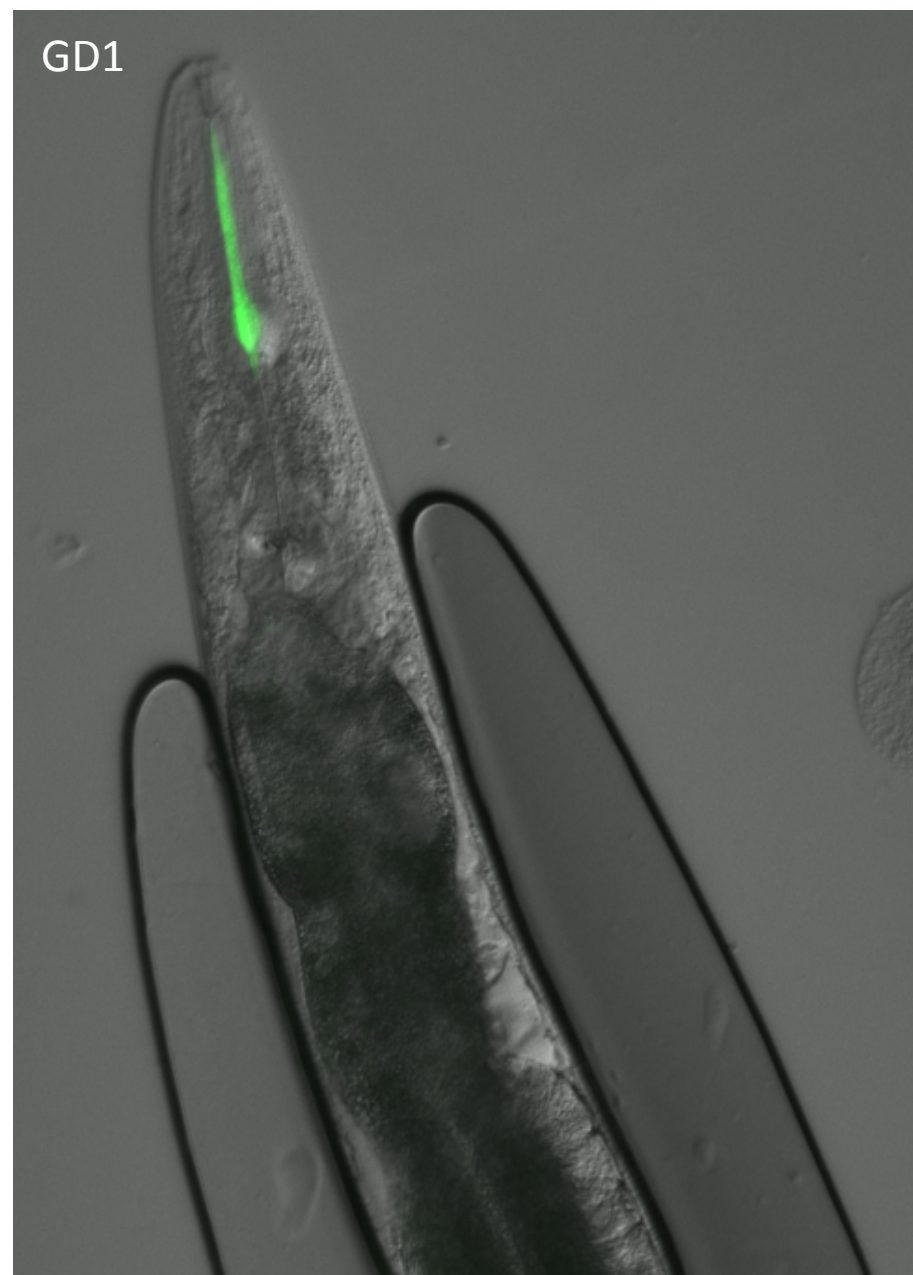

Supplement: Additional file 2 — Close-up view of day five adult worms fed OP50 or GD1 E. coli diets. Worms were fed OP50 or GD1 E. coli strains carrying a GFP-expressing plasmid from the hatchling stage and imaged at day five of adulthood. GFP-E. coli are evident as a large bolus in the anterior gut of the OP50-fed worm (left panel); GFP-E. coli are evident only in the anterior pharynx in the GD1-fed worm (right panel) (scale bar = 50 um). [file 1471-2180-12-300-S2.pdf]

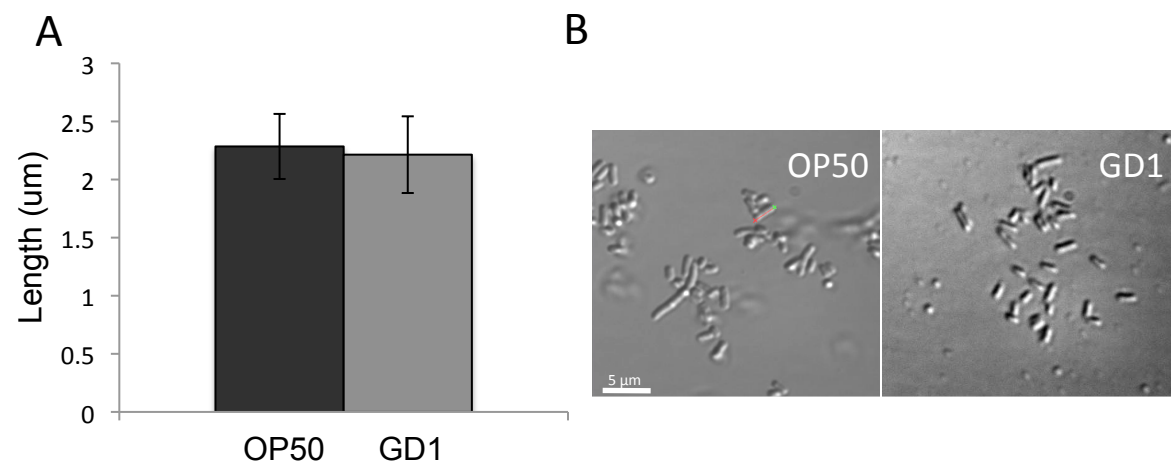

Fig S3

Supplement: Additional file 3 — GD1 and OP50 E.coli are similar in size. OP50 and GD1 E. coli cultures were grown overnight and visualized as described in Methods and Materials. Fifteen cells were measured per strain. The line traversing the cell in the OP50-panel demonstrates the dimension measured. Data subjected to Student’s t-test at a significance level of p < 0.05. [file 1471-2180-12-300-S3.pdf]
